# A Median-Ratio Scene-Based Non-Uniformity Correction Method for Airborne Infrared Point Target Detection System

**DOI:** 10.3390/s20113273

**Published:** 2020-06-08

**Authors:** Shuai Ding, Dejiang Wang, Tao Zhang

**Affiliations:** 1 Key Laboratory of Airborne Optical Imaging and Measurement, Changchun Institute of Optics, Fine Mechanics and Physics, Chinese Academy of Sciences, Changchun 130033, China; dingshuai@ciomp.ac.cn; 2 University of Chinese Academy of Sciences, Beijing 100084, China; ZhangT@ciomp.ac.cn; 3 Light Publishing Group, Changchun Institute of Optics, Fine Mechanics and Physics, Chinese Academy of Sciences, Changchun 130033, China; 4 Changchun Institute of Optics, Fine Mechanics and Physics, Chinese Academy of Sciences, Changchun 130033, China

**Keywords:** infrared detector, small target detection, non-uniformity, median-ratio scene-based NUC

## Abstract

Infrared detectors suffer from severe non-uniform noise which highly reduces image resolution and point target signal-to-noise ratio. This is the restriction for airborne point target detection systems in reaching the background limit. The existing methods are either not accurate enough, or too complex to be applied to engineering. To improve the precision and reduce the algorithm complexity of scene-based Non-Uniformity Correction (NUC) for an airborne point target detection system, a Median-Ratio Scene-based NUC (MRSBNUC) method is proposed. The method is based on the assumption that the median value of neighboring pixels is approximately constant. The NUC coefficients are calculated recursively by selecting the median ratio of adjacent pixels. Several experiments were designed and conducted. For both the clear sky scene and scene with clouds, the non-uniformity is effectively reduced. Furthermore, targets were detected in outfield experiments. For Target 1 48.36 km away and Target 2 50.53 km away, employing MRSBNUC the SNR of the target increased 2.09 and 1.73 times respectively compared to Two-Point NUC. It was concluded that the MRSBNUC method can reduce the non-uniformity of the detector effectively which leads to a longer detection distance and fewer false alarms of the airborne point target detection system.

## 1. Introduction

Airborne infrared small target detection systems are widely used in military fields for they possess night vision and anti-hidden capability, as well as mist-penetrating power. Usually, targets need to be detected as far away as possible for early warning. They are tiny and appear as dim point targets on the focal plane. 

For airborne detection systems, the complex aerial imaging environment seriously affects imaging quality and reduces detection rate. First, infrared radiation is easily affected by atmospheric attenuation, such as the absorption of atmospheric gas molecules, the scattering of suspended particles in the atmosphere, and the blocking effect under meteorological conditions. Second, both the target and the detection system are exposed to the air, the weather, season, day and night, and clouds all affecting imaging quality. Third, the imaging equipment is installed on various unmanned aerial vehicles (UAV) or manned aircraft. Vibration during in-flight imaging severely undermines imaging resolution. All those factors may cause infrared radiation in some scenes or regions of the infrared image to exceed the infrared radiation intensity of the small target local area, so that the target is drowned in a complex background. As a result, it has been a challenging task to detect small targets under a complex background [1,2,3]. 

Infrared detectors are often subject to severe non-uniform noise due to factors such as semiconductor materials, fabrication processes, readout circuits, and amplifier circuits [4,5]. Non- uniform noise seriously affects the imaging quality of the system, reduces the system resolution and the point target Signal-to-Noise Ratio (SNR), which is the bottleneck restricting the infrared point target detection system to reach the background limit [6,7]. Reducing non-uniform noise is an urgent problem to be solved for infrared point target detection systems [8]. 

In response to the non-uniformity problem of infrared detectors, scholars both at home and abroad have conducted relevant research and made some progress. At present, there are two major categories of calibration methods [9]: calibration-based [10,11,12] and scene-based [13,14,15] methods. The most commonly used calibration method is the two-point Non-Uniformity Correction (TPNUC) method. However, it cannot meet the high precision requirements of airborne early warning systems for the following reasons: (1) The radiation response of long-wave infrared detectors drifts slowly over time; (2) It is impossible to simulate complex airborne environments in the laboratory effectively. In order to compensate for the infrared detectors drift with time, an on-board embedded blackbody calibration method has been proposed in engineering [16]. Limited to the carrier‘s volume and weight, the blackbody can only be embedded in the front of the second mirror of all the optical lenses, so the main mirror is excluded in the calibration. The results are not reliable. Besides, the imaging process needs to be stopped during calibration which does not meet the efficiency requirement. 

There are two types of scene-based NUC methods: (1) The statistics-based method [13,17,18,19,20], which relies on the spatial-temporal assumption and completes the NUC process by adjusting the factors. Typical statistical methods include the constant statistical [13,17,18], neural network, and Kalman filter method [19,20], etc. The disadvantage of this type of method is that some application scenarios have difficulty in meeting the assumptions of such methods, and it is easy to produce ghost phenomenon. (2) The registration-based method [21,22,23,24,25], which assumes that different pixels respond identically for the same scene point within certain blocks of time. However, this type of method requires a complex registration algorithm, and the error is easily accumulated and spread, which is challenging to implement in engineering.

This research focuses on improving the precision and reducing the algorithm complexity of scene-based NUC in airborne infrared early warning systems. A strategy and a method of statistical scene-based NUC (Median-Ratio Scene-Based NUC) are proposed. The method is based on the assumption that for infrared detector arrays neighboring pixels should respond similarly within the exposure time. The effectiveness of the proposed method is then verified in simulation and practical application in the point target detecting system.

The rest of the paper is organized as follows: Section 2 introduces the proposed method and how to evaluate the effect after applying the method. Experimental details of the proposed method and comparison method are also described. Section 3 presents the comparison results of experiments. Method design considerations, results interpretation, and future research are discussed in Section 4. Section 5 concludes the research work.

## 2. Materials and Methods

### 2.1. Bad Points Replacement 

The existence of bad points will cause fixed white and black points to appear when imaging with an infrared focal plane array, which will seriously affect the visual effect of the image. Therefore, when using infrared focal plane arrays, the blind points must be processed.

#### 2.1.1. Bad Pixels Detection Algorithm Based on the Sliding Window 

The response rate of each pixel B_*i*,*j*_ is defined as the time-domain average of the gray value of each pixel [26]:

(1) $$B_{i,j}=\frac{\sum_{n=1}^{k}R_{i,j}\left(n\right)}{k}$$

where $R_{i,j}\left(n\right)$ is the gray value for the (*i*,*j*)th pixel of n-th frame, *k* is the total frames that are used for calculation, here we set *k* = 10 due to engineering experience. 

Query the average gray value of all pixels in the 3 × 3 sliding window to find the maximum and minimum pixel gray value: *B_max_* and *B_min_*. Remove the maximum and minimum values, and find the average value $\bar{B}$ of the remaining pixel gray levels in the window, that is,

(2) $$\bar{B}=\frac{\sum_{i=1}^{3}\sum_{j=1}^{3}B_{i,j}-B_{max}-B_{min}}{3\times 3-2}$$

Calculate the percentage of $\left(B_{max}-\bar{B}\right)$ and $\left(\bar{B}-B_{min}\right)$ relative to $\bar{B}$:

(3) $$\Delta =\frac{B_{max}-\bar{B}}{\bar{B}}\;\mathrm{or}\;\Delta =\frac{\bar{B}-B_{min}}{\bar{B}}$$

when Δ ≥ 10%, the pixel is considered to be a bad point. Set the blind element position (*i*,*j*) corresponding to the position element in the infrared focal plane array to 1. Set the remaining positions to 0. This matrix can be called a bad point matrix.

#### 2.1.2. Bad Points Compensation: Neighborhood Substitution

The blind pixel *P_i,j_* detected in the original image of the current frame is replaced by the mean value of the four pixels adjacent to the blind pixel. The replacement formula is:

(4) $$P_{i,j}=\frac{P_{i-1,j}+P_{i+1,j}+P_{i,j-1}+P_{i,j+1}}{4}$$

### 2.2. The Observation Model of Median-Ratio Scene-Based NUC (MRSBNUC) 

Due to observation, the model of calibration can be approximately expressed as a simple linear model:

(5) $$X_{i,j}\left(n\right)=v_{i,j}\left(n\right)\cdot R_{i,j}\left(n\right)+o_{i,j}\left(n\right)\;$$

where $X_{i,j}\left(n\right)$ and $R_{i,j}\left(n\right)$ are the gray values for the (*i*,*j*)th pixel of the n-th frame after correction and before correction respectively, $v_{i,j}\left(n\right)$ and $o_{i,j}\left(n\right)$ are the gain and offset parameters respectively. For an infrared Focal Plane Array (FPA) with *M* lines and *N* rows, i∈[1,M],j∈[1,N].

According to Equation (5), there are two parameters that need to be solved for an NUC method, which is easy for laboratory correction. However, it is not the case for scene-based NUC due to the difficulty of finding two effective constraints. So it can be simplified to a one-point model with the more dominant coefficient $v_{i,j}\left(n\right)$:

(6) $$X_{i,j}\left(n\right)=v_{i,j}\left(n\right)\cdot R_{i,j}\left(n\right)$$

Thus it is possible to discuss the problem of NUC coefficient $v_{i,j}\left(n\right)$ solving.

### 2.3. MRSBNUC Method

In order to solve the influence of the detector drift over time, it is necessary to study the method without pre-stored coefficients. This is really important for accurate in-flight correction. The MRSBNUC method proposed here is an in-flight correction method that can be performed anytime during the flight or imaging process without stopping the camera. It relies on the empirical observation that adjacent pixels of airborne infrared search and track (IRST) systems usually image similar scenes the same as one another [27]. Thus we make the assumption that the median value of neighboring pixels is approximately constant. 

**Figure 1:**
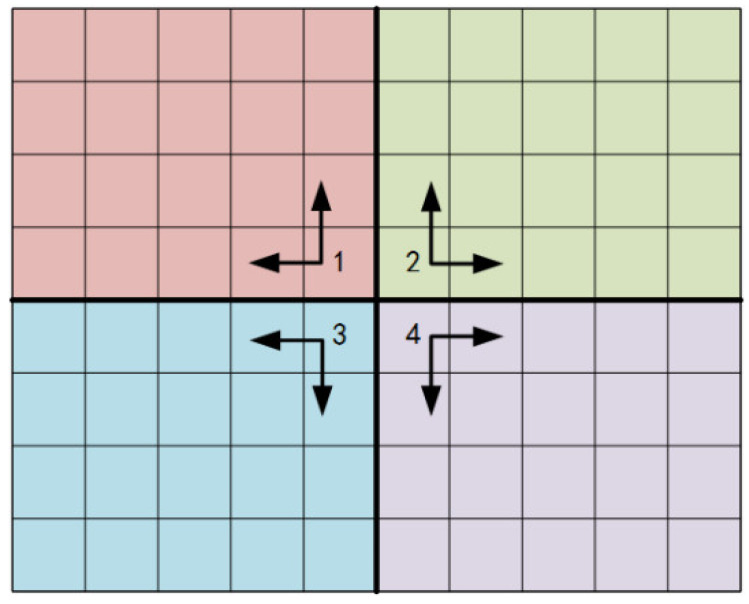
Adjacent pixels schematic.

For a pixel, neighboring pixels here mean two adjacent pixels: one from the horizontal direction and one from the vertical direction representatively. Taking the middle point of FPA as the original starting point, the FPA can be divided into four individual areas (Figure 1). For Section 1, the starting point is at the bottom right corner. The adjacent pixels are in the up and left side of the point recursively. The other three parts are the same. Take Section 2 as an example, this can be expressed as:

(7) $$median_{x}\left(\frac{X_{i,j}}{\sqrt{X_{i,j-1}\cdot X_{i-1,j}}}\right)=1$$

Substituting Equation (6) into Equation (7) to obtain:

(8) $$median_{x}\left(\frac{v_{i,j}\left(n\right)\cdot R_{i,j}\left(n\right)}{\sqrt{\left(v_{i,j-1}\left(n\right)\cdot R_{i,j-1}\left(n\right)\right)\cdot \left(v_{i-1,j}\left(n\right)\cdot R_{i-1,j}\left(n\right)\right)}}\right)=1$$

Since the parameter $v_{i,j}\left(n\right)$ is irrelevant with the frame number *n*, Equation (8) can be rewritten as:

(9) $$\frac{v_{i,j}}{\sqrt{v_{i,j-1}\cdot v_{i-1,j}}}median_{x}\left(\frac{R_{i,j}\left(n\right)}{\sqrt{R_{i,j-1}\left(n\right)\cdot R_{i-1,j}\left(n\right)}}\right)=1$$

For a sequence of camera frames (*n* frames) which is considered as *n* samples in the method, compute the ratios between adjacent pixels, $r_{i,j}=\frac{R_{i,j}}{\sqrt{R_{i,j-1}\cdot R_{i-1,j}}}$ (where i∈[2,M],j∈[2,N]). Then the median ratio of $r_{i,j}$ can be expressed as ${\tilde{r}}_{i,j}$, NUC can be calculated with:

(10) $$v_{i,j}=\frac{\sqrt{v_{i,j-1}\cdot v_{i-1,j}}}{{\tilde{r}}_{i,j}}$$

**Figure 2:**
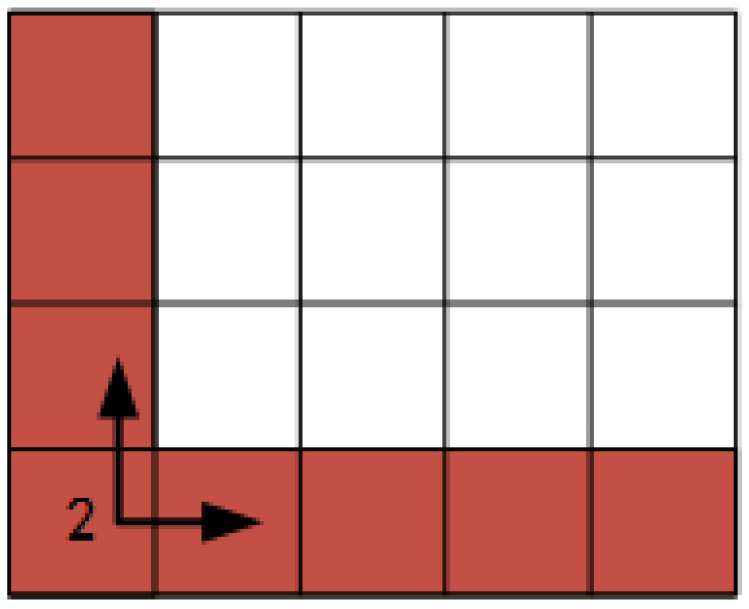
The problem is transferred to solving the Non-Uniformity Correction (NUC) for the first row and first column of the section.

It is obvious as seen in Equation (10) that the solution of $v_{i,j}$ is iterative. That is to calculate $v_{i,j}$ we need to know $v_{i,j-1}$ and $v_{i-1,j}$ in advance. This process eventually leads to the problem of getting the parameters of $v_{i,1}$ and $v_{1,j}$ which are the NUC coefficient for the first row and first column (Figure 2, taking Section 2 as an example). Thus the problem of solving NUC for detector arrays becomes that of solving NUC for two linear detectors. 

For linear arrays, we continue to implement the MRSBNUC method. The difference between linear array and area array is that the adjacent pixel is the one pixel next to it in one direction. Take the first row (where *j* is set to 1) as an example to illustrate this method for linear arrays. It is defined:

(11) $$median_{x}\left(\frac{X_{i,1}}{X_{i-1,1}}\right)=1$$

Accordingly, the same as Equation (9), we can get:

(12) $$\frac{v_{i,1}}{v_{i-1,1}}median_{x}\left(\frac{R_{i,1}\left(n\right)}{R_{i-1,1}\left(n\right)}\right)=1$$

The ratio between adjacent pixels is computed by:

(13) $$r_{i,1}=\frac{R_{i,1}}{R_{i-1,1}}$$

Select the median ratio of $r_{i,1}$ for linear array which is expressed as ${\tilde{r}}_{i,1}$. It is derived from Equation (12) and Equation (13):

(14) $$v_{i,1}=\frac{v_{i-1,1}}{{\tilde{r}}_{i,1}}\;\;\left(i\in \left[2,M\right]\right)$$

Similarly, for the first column, the non-uniformity correction coefficient can be calculated by the following formula:

(15) $$v_{1,j}=\frac{v_{1,j-1}}{{\tilde{r}}_{1,j}}\;\left(j\in \left[2,N\right]\right)$$

One more thing that needs to be pointed out is that it can be seen from Formula (12) that the number of unknowns is *M*, and there are *M*–1 independent equations, which results in a non-unique solution for this equation set. It is the uniformity that matters the most in this method, so we initialize the *v*_1, 1_ = 1 to get a unique solution of the formula. 

**Figure 3:**
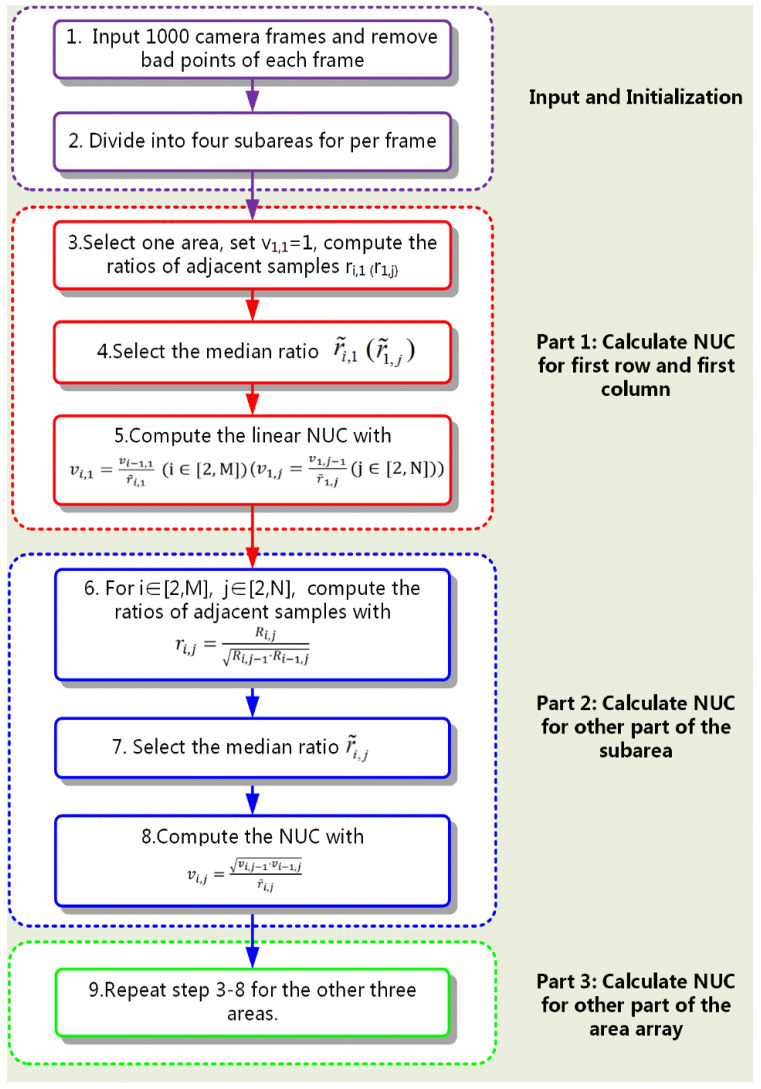
Procedure of MRSBNUC method.

To sum up, the procedure of the MRSBNUC method is shown in Figure 3. As can be seen from Figure 3, the method requires 1000 frames of the camera, simple multiplication and division operations, and calculates only one parameter of NUC—only the gain. Compared to traditional scene-based methods [17,18,19,20,21,22,23,24,25] which need to calculate two parameters (both the gain and the bias), and require 10,000 images or a complicated motion estimation or registration algorithm, the MRSBNUC method reduces the complexity of scene-based NUC significantly.

### 2.4. Uniformity Evaluation

Uniformity can usually be characterized by the global standard deviation (STD) of the gray value of the detector. However for an IRST, especially for the point target detecting, it is the SNR of the target that decides whether the target can be detected. Therefore global uniformity is not as effective as local STD. So local STD (5 × 5) $\sigma_{l}$ is used for uniformity evaluation. It is defined as:

(16) $$\sigma_{l}=\sqrt{\frac{1}{5\times 5}\overset{1}{\underset{x=-1}{\sum }}\overset{1}{\underset{y=-1}{\sum }}{\left(R_{i+x,j+y}-\mu \right)}^{2}}\;\;\;\;\;\left(i\in \left[2,M-1\right],\;j\in \left[2,N-1\right]\right)$$

where *μ* is the arithmetic mean of the 5 × 5 pixels:

(17) $$\mu =\frac{\sum_{x=-1}^{1}\sum_{y=-1}^{1}R_{i+x,j+y}}{5\times 5}$$

### 2.5. Experiments

To verify the effectiveness of the proposed NUC method, experiments need to be designed. The two-point NUC calibration (TP) method is commonly used in engineering. We conducted the laboratory calibration for comparison and basic effective foundation. 

#### 2.5.1. Laboratory Calibration

A FLIR HgCdTe Long-Wave Infrared (LWIR) detector was used in the experiments. The parameters of the IR system are listed in Table 1. 

**Figure 4:**
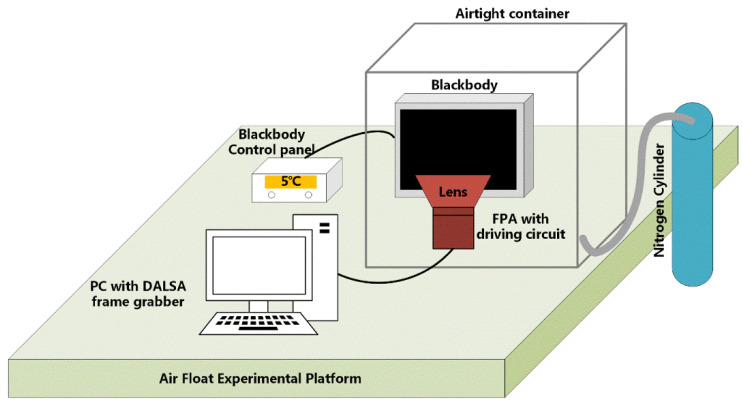
Schematic diagram of the laboratory calibration experiment.

The schematic diagram of the laboratory calibration experiment is shown in Figure 4. All devices are placed on the vibration isolation platform. A CI blackbody with a controller is located right in front of the IR system (infrared detector, optical lens, and driving circuits). The camera is linked to a DALSA frame grabber connected to a PC through a camera link cable. 

**Figure 5:**
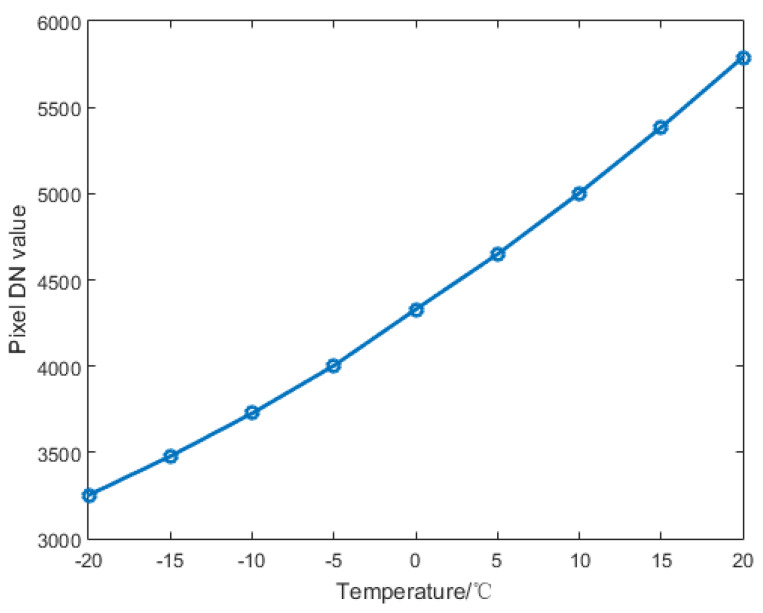
Relationship between Digital Number (DN) value of the image and the black body temperature.

The integration time of the IR system is set to 300 μs. By setting the black body temperature from −20 to +20 °C at intervals of 5 °C, we can adapt the incident radiance into the IR detector. Image data for each temperature is restored in the PC through the grabber. The relationship between the average gray value of the image and the black body temperature is shown in Figure 5.

The TP NUC method was applied to analyze data collected in the laboratory. Since it was a laboratory test, we could use the global STD to indicate the uniformity of the IR system. The images used to calculate the NUC parameters are marked as R1 and R2. The corresponding temperature of the blackbody is T1 and T2, respectively. The image needing to be corrected is labeled R3. The corresponding temperature is T3. Two different TP NUC experiments were conducted:

- Experiment 1: T3 Falls Outside the Range of T1 and T2

**Figure 6:**
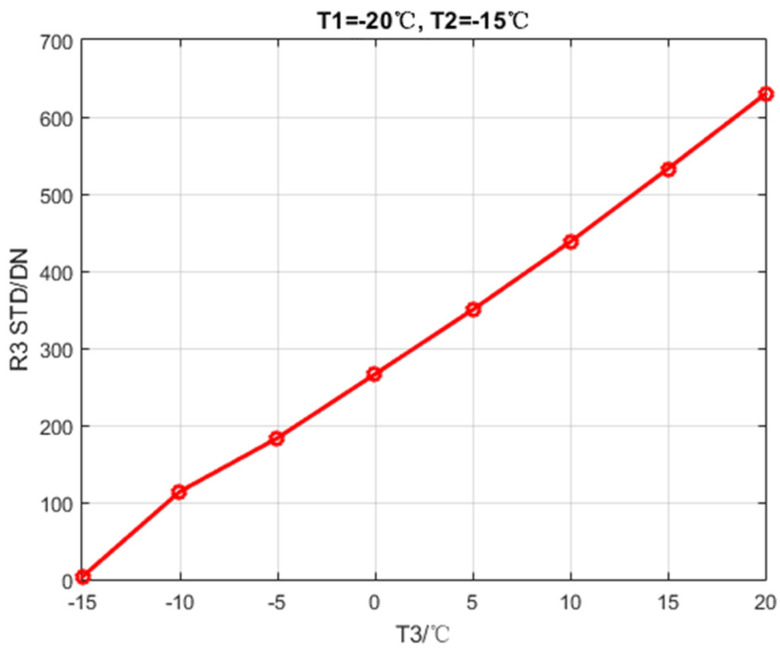
Global STD after NUC changes with T3.

Set T1 to −20 °C, T2 to −15 °C, T3 increases from −15 to 20 °C at intervals of 5 °C. Then calculate the global STD of R3. The calibration data are listed in Table 2. The global STD after NUC is shown in Figure 6.

- Experiment 2: T3 Falls between T1 and T2

**Figure 7:**
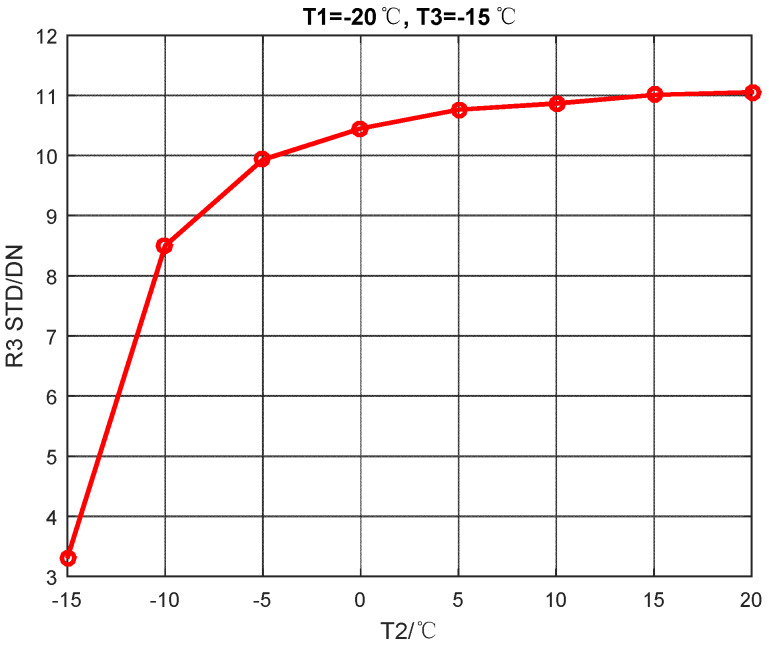
Global STD after NUC changes with T2.

Set T1 to −20 °C, T3 to −15 °C, T2 increases from −15 to 20 °C at intervals of 5 °C. Then calculate the global STD of R3. The calibration data is listed in Table 3. The global STD after NUC is shown in Figure 7. 

#### 2.5.2. Scene-Based Experiment

**Figure 8:**
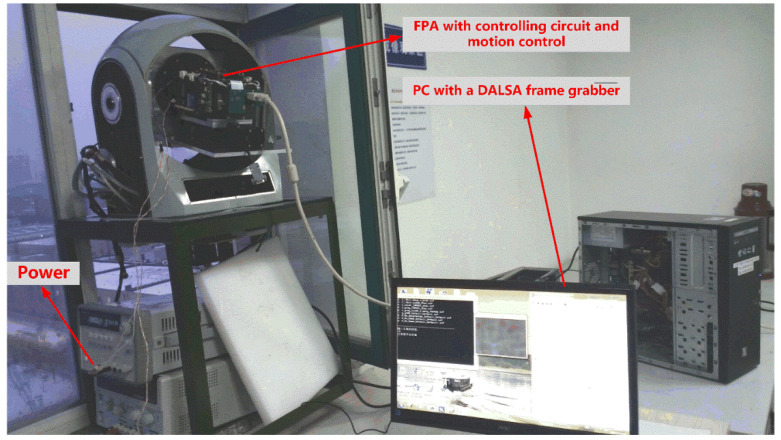
Experimental devices of scene-based NUC calibration.

In order to verify the validity of the method, an optical imaging platform was set up to integrate the long-wave infrared camera with the turntable system. Except for collecting image data, an industrial computer was also used to control the camera speed and rotation direction through the RS422 serial port to simulate the working mode of the detection system. The integration time was adjusted to 300 us, which is consistent with the laboratory calibration to facilitate the comparison of the effects of two NUC methods. The actual experimental platform is shown in Figure 8.

The experimental process is as follows: First, adjust the camera height and elevation angle to align it with the scene outside the window. Second, change the azimuth of the camera at a constant speed to simulate the working mode of the airborne infrared point target detection system: sweeping mode, and then start the camera image acquisition process. The scene is continuously collected through the CameraLink interface, and more than 1500 sequence image raw data are stored in the computer.

To exclude the influence of infrared detector drift over time, weather, season, and other factors on the experimental results, the two experiments, laboratory calibration and scene-based experiment were performed in the same week. Since the IRST is for air-to-air small target detection, it is mainly focused on the sky scene background. Two different sets of scene image data were stored: clear sky scene and sky scene with clouds. The collected scene image data is corrected using the TP method and the MRSBNUC method proposed in this paper, and the correction results are compared and analyzed. 

#### 2.5.3. Target Detection

**Figure 9:**
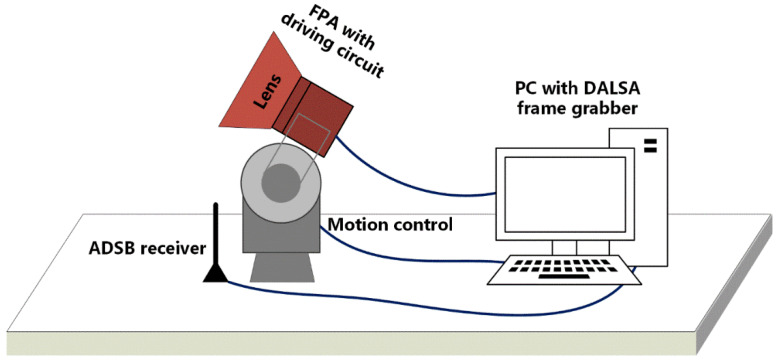
Schematic diagram of the target detection experiment.

In order to verify the effectiveness of this method in actual target detection, an experiment to detect the airliners leaving our local International Airport was designed. The schematic diagram of the experimental platform is shown in Figure 9. The IR imaging system was mounted to a motion control system so that the angle of the optical window could be adjusted due to requirements. A global navigation system (GNS 5890 ADSB receiver) was placed as near as possible to the IR target imaging system. An ADSB receiver was used to assist us in finding the airliners (actual targets) manually in the image frames sequence. The distance from the imaging system and the target can also be calculated with data read from the ADSB receiver. The imaging system, motion control system, and ADSB receiver are connected to a PC through CameraLink, Ethernet, and USB connectors separately. The whole devices were placed on the roof of a building. 

The experimental process is as follows: First, adjust the camera height and elevation angle to align it with the sky background scene and potential target passing area. Second, set up the ADSB receiver and show all the data (location, velocity, identification of the airliners, etc.) from ADSB Scope software on the PC screen. Third, change the azimuth of the camera at a constant speed to simulate the working mode of the airborne infrared point target detection system—sweeping mode— and then start the camera image acquisition. The scene is continuously collected through the CameraLink interface, and images of raw data are stored in the computer. Fourth, find the frames with airliners in the Field of View(FOV). Fifth, calculate the SNR of the target both after TPNUC and MRSBNUC. 

## 3. Results

### 3.1. Clear Sky Scene

**Figure 10:**
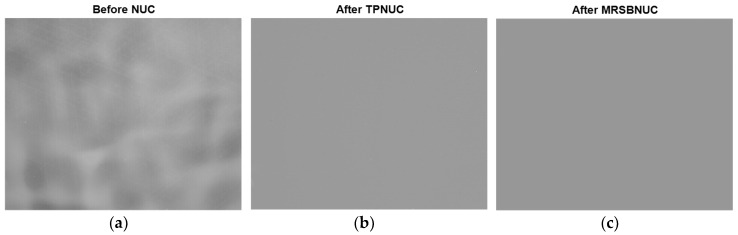
Clear sky scenes: (**a**) before NUC, (**b**) after TPNUC and (**c**) after MRSBNUC.

Take one frame as an example, the original and corrected images are shown in Figure 10. As can be seen from Figure 10a, the original image has strong non-uniformity, which causes the image to be uneven. Both the TPNUC and the MRSBNUC method can improve the uniformity of the original image effectively. Visually, there is no ghosting phenomenon in the corrected images. However, there is still some noise remaining in Figure 10b, and the correction effect of Figure 10c is better than Figure 10b. 

**Figure 11:**
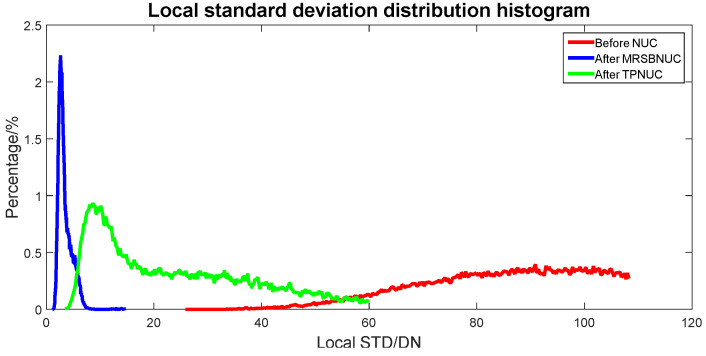
Local standard deviation distribution of clear sky scene: before NUC, after TPNUC, and after MRSBNUC.

**Figure 12:**
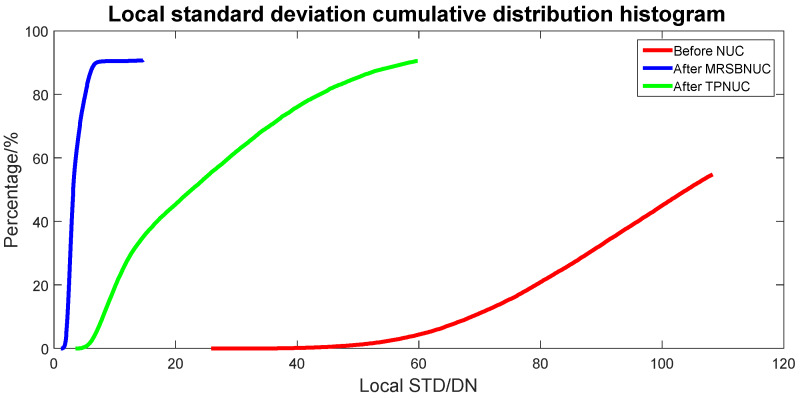
Local standard deviation cumulative distribution of clear sky scene: before NUC, after TPNUC, and after MRSBNUC.

To evaluate the correcting effect quantitatively, local (5 × 5 pixels) STD distributions and local (5 × 5 pixels) STD cumulative distributions are calculated and shown in Figure 11 and Figure 12. The 5 × 5 neighborhood local STD means of the scene before NUC, after TPNUC, and after MRSBNUC are 109.8, 39.9, and 5.2 separately. It is indicated that for clear sky scenes MRSBNUC is much better than TPNUC. 

### 3.2. Sky Scene with Clouds

**Figure 13:**
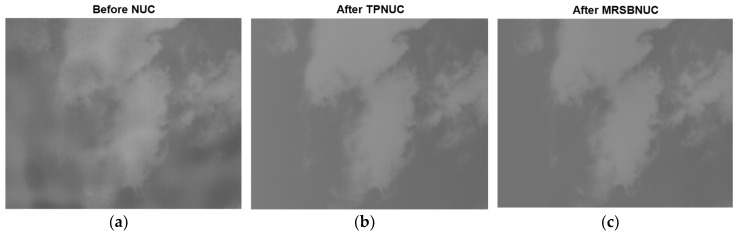
Sky scene with clouds: (**a**) before NUC, (**b**) after TPNUC and (**c**) after MRSBNUC.

For the sky scene with clouds, also take one frame as an example, the original and corrected images are shown in Figure 13. The same as in Figure 10, it can be seen from Figure 13a, the original image has strong non-uniformity, which causes the image to be uneven. Both the TPNUC and the MRSBNUC methods can improve the uniformity of the original image effectively. Visually, there is no ghosting phenomenon in the corrected images. However, there is still some noise remaining in Figure 13b, and the correction effect of Figure 13c is better than Figure 13b. 

**Figure 14:**
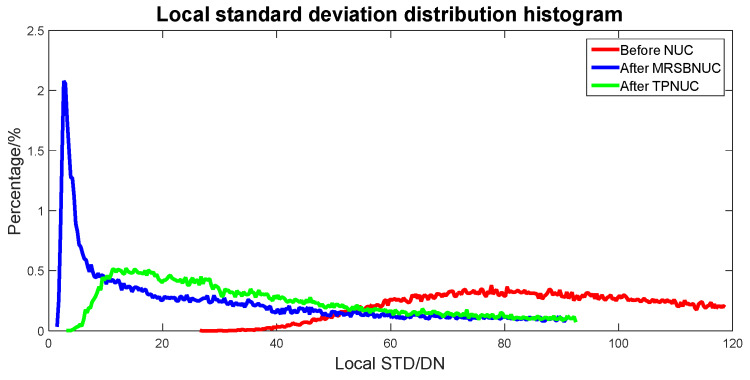
Local standard deviation distribution of sky scene with clouds: before NUC, after TPNUC, and after MRSBNUC.

**Figure 15:**
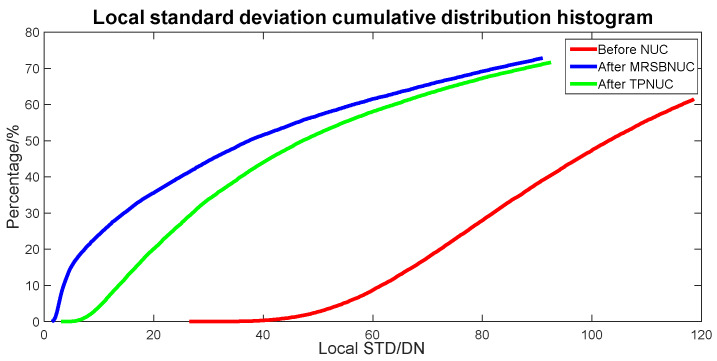
Local standard deviation cumulative distribution of sky scene with clouds: before NUC, after TPNUC, and after MRSBNUC.

To evaluate the correcting effect quantitatively, local (5 × 5 pixels) STD distributions and local (5 × 5 pixels) STD cumulative distributions are calculated and shown in Figure 14 and Figure 15. The 5 × 5 neighborhood local STD means of scene before NUC, after TPNUC, and after MRSBNUC are 118, 75.4 and 62.3 separately. It is indicated that for sky scene with clouds MRSBNUC is also better than TPNUC. 

### 3.3. Target Detection

**Figure 16:**
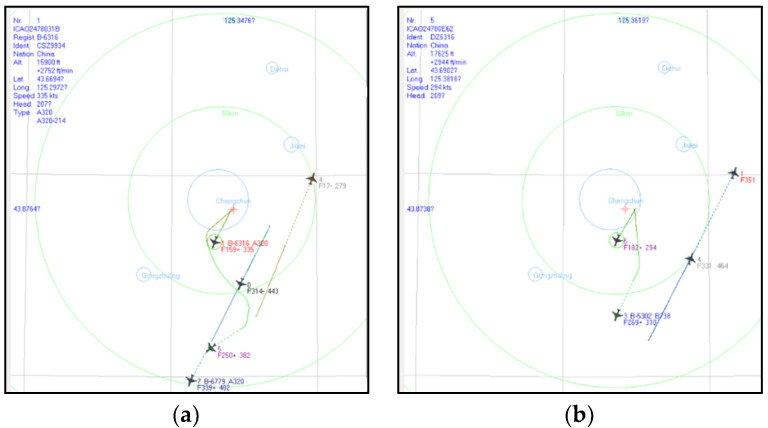
ADSB receiver data shown on ADSB Scope Software: (**a**) Target 1 Airline 0 from Korea was detected; (**b**) Target 2 Airline 4 from China was detected.

Figure 16 is the screenshot of ADSB Scope Software. Targets could be found manually based on the ADSB data. The distance between one target and the IR system can also be calculated employing the geodetic coordinate system. The experiment was done repeatedly on different dates, and some targets were captured. In order to show the effectiveness of the algorithm, among those targets two representative targets in the cloudy scene were selected to introduce the experimental results. In Figure 16a, Target 1 Airline 0 from Korea was detected. It is 48.36 km away from the IR system. In Figure 16b, Target 2 Airline 4 from China was detected. It is 50.53 km away from the IR system.

**Figure 17:**
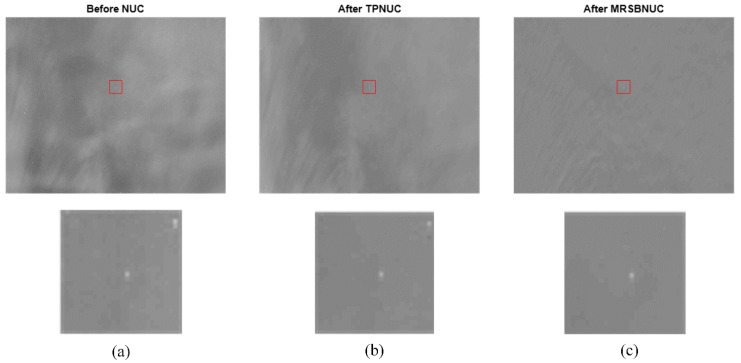
Target 1: (**a**) before NUC, (**b**) after TPNUC and (**c**) after MRSBNUC.

**Figure 18:**
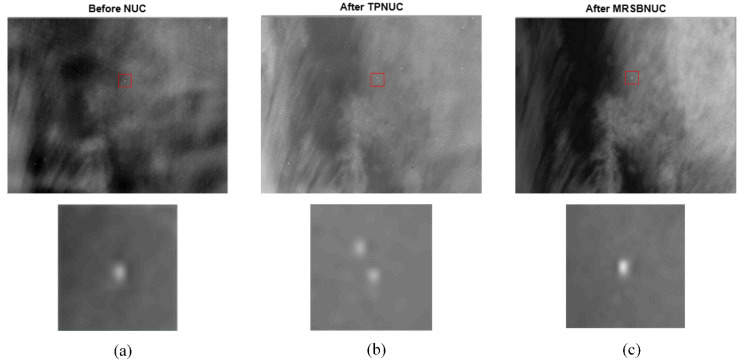
Target 2: (**a**) before NUC, (**b**) after TPNUC and (**c**) after MRSBNUC.

For scenes with the target in FOV, also take one frame as an example, the original and corrected images of Target 1 and Target 2 are shown in Figure 17 and Figure 18. The 5 × 5 neighborhood local STD means of the scene before NUC, after TPNUC and after MRSBNUC are 118.2, 78.2, and 52.6 separately in Figure 17. Local STD means increased by 1.49 times; In Figure 18, The 5 × 5 neighborhood local STD means of scene before NUC, after TPNUC and after MRSBNUC are 123.5, 80.1, and 52.4 separately. Local STD means increased by 1.53 times. 

Signal-to-Noise (SNR) is often used for evaluation of detection results. Image SNR is defined as [28,29]:

(18) $$\mathrm{SNR}=\frac{\left|U_{t}-U_{b}\right|}{\sigma_{b}}$$

where $U_{t}$ and $U_{b}$ are the gray value of the detection unit corresponding to the target and local background area respectively, $\sigma_{b}$ is the noise standard deviation of the local background. It can also be written in decibels:

(19) $$\mathrm{SNR}\left(\mathrm{dB}\right)=10\log_{10}\left(\frac{\left|U_{t}-U_{b}\right|}{\sigma_{b}}\right)$$

For an infrared point target, Signal-to-Noise can be calculated with [28]:

(20) $$\mathrm{SNR}=10\mathrm{lg}\frac{I_{max}-B}{\sigma_{\mathrm{noise}}}$$

where $I_{max}$ is the gray value of target, B is the mean gray value of the background area, and $\sigma_{\mathrm{noise}}$ represents noise standard deviation. 

The target SNR before NUC, after TPNUC and MRSBNUC for Target 1 and Target 2 are listed in Table 4. As can be seen from the results in Table 4. For Target 1, the SNR is increased by 1.88 and 3.93 times after TPNUC and MRSBNUC, respectively. For Target 2, the SNR is increased by 1.60 and 2.76 times after TPNUC and MRSBNUC, respectively. Compared to TPNUC that is usually used in engineering, utilizing MRSBNUC target SNR is increased by 2.09 times and 1.73 times for Target 1 and Target 2. Therefore, the performance of MRSBNUC is better than TPNUC in target detection. 

## 4. Discussion

### 4.1. Why Does the MRSBUNC Method Calculate from the Center of the Image (Divided into Four Regions)?

As is shown in Equation (10), this method is based on a recursive process. The error and uncertainty will also be accumulated and passed on during the calculation. We start the NUC calculation from the center of the image so as to limit the error accumulation. Even with this strategy the parameter $v_{i,j}$ could slowly change from the center to the edges of one picture due to statistical noise among thousands of samples. It means that the NUC can be accurate locally and inaccurate globally which depends on the data collected. Since we are trying to improve the detecting rate of the point target in this paper, the accurate local NUC is good enough and practical. For scenes that require high global uniformity, this method may not be effective.

### 4.2. Interpretation of Results

The range R of small target detection based on signal-to-noise ratio is calculated with:

(21) $$R=\sqrt{\frac{D^{*}A_{t}A_{0}\tau \left(R\right)\left(L_{t}-L_{b}\right)}{{\left(A_{d}\cdot \Delta f\right)}^{1/2}\cdot \left(SNR_{inf}\right)}}$$

where $D^{*}$ is the specific detection rate of a detector, $A_{t}$, $A_{0},$ and $A_{d}$ are the areas of target radiation, the entrance pupil and detector array respectively, Δf is the effective noise bandwidth of the detector’s amplifier, τ(R) is the atmospheric spectral transmittance, $L_{t}$, $L_{b}$ are radiances of the target and the uniform background respectively. $SNR_{inf}$ is the limit of signal-to-noise ratio required under certain detection probability. 

From Section 3 we can see that the MRSBNUC results of both the clear sky scene and sky scene with clouds are better than that of TPNUC which is often used in engineering. For the clear sky scene the uniformity (local STD) mean decreased from 39.9 to 5.2, which was reduced by 7.7 times. As shown in Table 2 and Table 3, the background limit is 3.3 (both the calculating and correcting temperature are −15 °C). It should be noticed that after MRSBNUC, the uniformity of the image approaches its background limit. As is indicated in Equation (21), accordingly, the SNR is increased to 59.3 and the detection range is increased to 7.7 times. 

For cloudy scenes, the uniformity (local STD) mean decreased from 75.4 (TPNUC) to 62.3 (MRSBNUC), decreased by 1.2 times. The corresponding SNR is increased to 1.46 times, with a nearly 50% improvement rate. According to Equation (21), the detection distance is 20% increased. It is clear that for scenes with heavy clouds, although the NUC effect is not as good as the clear sky scene, it still improves the target detection range and reduces the false alarm rate. In summary, the detection distance of the system can be increased by 1.2–7.7 times due to different sky scene backgrounds.

For the actual target detection experiment, as can be seen in Figure 17 and Figure 18, due to the difference in weather conditions and time at which the images were captured, the image could show differently. Take a close look at the figures. It can be discovered that the little blob in the upper right corner in the small picture of Figure 17a,b is not shown in Figure 17c. Also Figure 18b has one more blob except for the target, which is not shown in Figure 18a,c. These circumstances are due to TPNUC being based on laboratory calibration data. The detector response drifts over time, and it is hard to simulate the sky background environments precisely in the laboratory. So when employing TPNUC to actual target detection images, some noises and non-uniformity may not be corrected as in Figure 17b and noises could be introduced to the original image as in Figure 18b. These circumstances do not exist in Figure 17c and Figure 18c. This further strengthens the results that the MRSBNUC method has better performance than TPNUC.

Furthermore, the SNRs of two representative targets after MRSBNUC are increased by 2.09 times and 1.73 times compared to TPNUC, respectively. Accordingly, the detection range of these two actual targets can also be increased. It shows that the proposed method MRSBNUC can increase the uniformity of an IR image and the detection distance effectively. 

### 4.3. Future Research 

According to the results and discussion above, future research should be devoted to solving aspects such as error transfer and correction of heavy-cloud scenes. It is very important to find a universal scene correction method suitable for various scenes.

## 5. Conclusions

In this work, a Median-Ratio Scene-based NUC method was proposed for the airborne infrared point target detection system. The presented method is based on a simple assumption which is commonly acknowledged in digital images. It is a timely method which can be used anytime during the flight without stopping the imaging process. Experiments showed that the proposed method is of high ability in reducing the non-uniformity of target detection images which leads to higher target SNR and longer detection range. Except for a better correction effect, the proposed method also reduced the algorithm complexity significantly in terms of parameter number, algorithm process, and the amount of input data required, thus making it possible to be employed in engineering. 

## Figures and Tables

**Table 1:** Parameters of the experimental IR system.

Parameter	Value
Response band (μm)	7.7–9.3
Pixels	640 × 512
Pixel size (μm)	15
NEDT (mK)	20 mk@300 k@Ti = 300 μs
Camera output resolution (bit)	14
Operating temperature (K)	77
Full frame rate (f/s)	100
Focal length (mm)	60–300
F/#	4

**Table 2:** Two-point Non-Uniformity Correction (TP NUC) statistics of experiment 1.

T1 (°C)	T2 (°C)	T3 (°C)	Global STD after NUC
−20	−15	−15	3.30
−20	−15	−10	113.93
−20	−15	−5	183.44
−20	−15	0	266.28
−20	−15	5	349.71
−20	−15	10	437.89
−20	−15	15	532.52
−20	−15	20	629.66

**Table 3:** TP NUC statistics of experiment 2.

T1 (°C)	T2 (°C)	T3 (°C)	Global STD after NUC
−20	−15	−15	3.30
−20	−10	−15	8.51
−20	−5	−15	9.93
−20	0	−15	10.44
−20	5	−15	10.76
−20	10	−15	10.86
−20	15	−15	11.00
−20	20	−15	11.05

**Table 4:** SNR before NUC, after TPNUC and MRSBNUC for Target 1 and Target 2.

Item	Target 1 SNR	Target 2 SNR	Target 1 SNR Increased by (Times)	Target 2 SNR Increased by (Times)
Before NUC	2.89	3.56	——	——
TPNUC	5.42	5.68	1.88	1.60
MRSBNUC	11. 37	9.83	3.93	2.76
